# Nitrogen-Enriched Cr_1−x_Al_x_N Multilayer-Like Coatings Manufactured by Dynamic Glancing Angle Direct Current Magnetron Sputtering

**DOI:** 10.3390/ma13163650

**Published:** 2020-08-18

**Authors:** Pedro Renato Tavares Avila, Alisson Mendes Rodrigues, Monica Costa Rodrigues Guimarães, Magdalena Walczak, Romualdo Rodrigues Menezes, Gelmires de Araújo Neves, Haroldo Cavalcanti Pinto

**Affiliations:** 1 São Carlos School of Engineering-EESC, University of São Paulo—USP, São Carlos-SP 13563-120, Brazil; pedro.avila@usp.br (P.R.T.A.); alisson.mendes@professor.ufcg.edu.br (A.M.R.); mony-rodrigues@uol.com.br (M.C.R.G.); 2 Materials Engineering Unit (UAEMa), Federal University of Campina Grande (UFCG), Campina Grande-PB 58109-970, Brazil; romualdo.menezes@ufcg.edu.br (R.R.M.); gelmires.neves@ufcg.edu.br (G.d.A.N.); 3 Department of Mechanical and Metallurgical Engineering, Escuela de Ingeniería, Pontificia Universidad Católica de Chile, Vicuña Mackenna, Santiago 4860, Chile; mwalczak@ing.puc.cl

**Keywords:** Cr-based coatings, multilayer, dcMS, DGLAD, stainless steel, surface modification

## Abstract

Multilayer-like CrN and Cr_1−x_Al_x_N coatings with different Al contents were deposited onto a stainless steel substrate using dynamic glancing angle deposition direct current magnetron sputtering (DGLAD dcMS) in a N rich atmosphere to understand the role of Al on the growth of the films and mechanical properties of the nitrides with a multilayer architecture. Chemical analysis by means of energy dispersive analysis (EDS) and glow discharge optical emission spectroscopy (GDOES) depth profiling revealed that while CrN samples were close to stoichiometric, the Cr_1−x_Al_x_N coatings presented excess N between 70 and 80% at. An expressive change in texture was observed as the CrN coating changed its preferred orientation from (111) to (200) with the addition of Al, followed by a modification in morphology from grains with faceted pyramidal tops in CrN to dome-shaped grains in Cr_1−x_Al_x_N coatings. Multilayer-like nanostructures of corrugated grains were produced with a periodicity of approximately 30 nm using dynamic glancing angle deposition. The deposition rate was drastically reduced with an increase of Al, meanwhile, the best mechanical performance was achieved for the coating with a higher content of Al, with hardness up to 27 GPa and a higher value of maximum resistance to plastic deformation.

## 1. Introduction

In the last decades, transition metal (TM) nitride films have been a field of intense study in surface science and technology. In this context, due to the versatility of manufacturing techniques, the properties of several ceramic systems (TiN, AlN, VN, NbN, ZrN, HfN, and CrN) have been evaluated. The wide variety of properties of this class of ceramic coatings has promoted their application and potential use in various areas, such as wear and corrosion protection, production of optical and magnetic devices, biomedical implants, and aesthetic layers, among others [1,2,3,4].

Reactive direct current magnetron sputtering (dcMS) is a prominent technique of thin-film production. Basically, in this technique, ions or neutrals are sputtered from a precursor target by a plasma of inert gas (Ar, mostly) entrapped in a magnetic field generated by a magnetron in the vicinity of the target. The sputtered species travel through the deposition chamber and condense on a substrate surface in the presence of a reactive gas (N_2_, CH_4_, etc.) [5]. Recently, a variation of conventional sputtering, namely dynamic glancing angle deposition (DGLAD), has been introduced, with continuous motion of the substrate as the film grows, affecting the angle of incidence of the sputtered material and changing the morphology of grains to a corrugated or undulated format [6,7]. It was reported that such oscillatory motion is capable of producing multilayer-like structures that can be tuned by the period and range of oscillation [8].

Reactive magnetron sputtering is subject to several control variables that can impact chemical composition, structure, and properties of the coating. Among these control variables, the reactive gas flow rate is an important parameter because it can determine the stoichiometry of the film. Indeed, reactive magnetron sputtering coatings with an excess of N can be manufactured under high N_2_ flow rates [9,10,11,12]. Nitrogen-rich CrN coatings have already been reported by [13,14,15], and their results showed that films with the N/TM ratio higher than 1 sometimes presented improved mechanical, optical, and magnetic properties as compared with their stoichiometric counterparts, indicating that N-rich TM nitrides could be an interesting choice in the engineering design of thin films.

TM nitride coatings have been improved even further by adding a ternary component to the system, as is the case for (Ti, Al)N and (Cr, Al)N [16]. Cr-based coatings are known to act as an important corrosion and oxidation barriers for metal substrates due to formation of Cr_2_O_3_ and other Cr-based phases that can passivate the surface, as described in the literature [17,18,19]. The production of ternary CrAlN has been reported to increase even more anticorrosive and oxidation protection, along with hardness and wear resistance [20,21]. In [22], it was reported that Al addition on CrN structure coatings affected their properties and the machining performance. In addition, nitrogen addition improved the life of the film based on wear and turning tests.

Therefore, this study aims to understand the effects of the Al/Cr ratio on the growth and mechanical properties of multilayer-like coatings of Cr-Al-N prepared by reactive magnetron sputtering with excess N content by fixing a high N_2_ flow rate (50 sccm) in the reaction chamber and changing the precursor target Al/Cr content. To our knowledge, this is the first work to address this subject.

## 2. Materials and Methods

### 2.1. Manufacturing of the Cr-Al-N Ceramic Coatings

**Figure 1:**
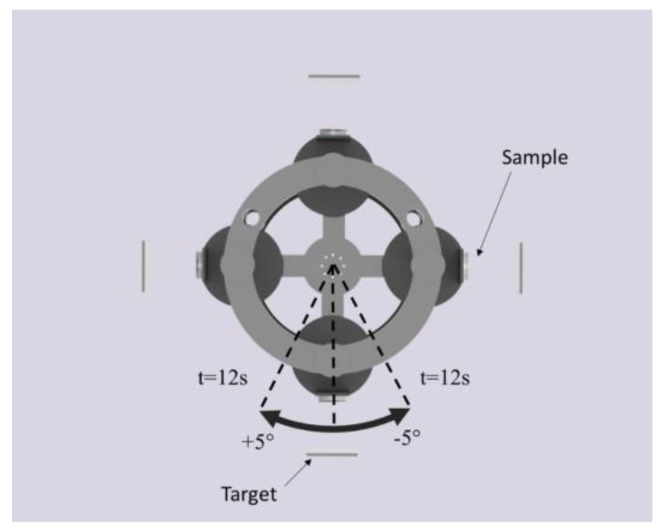
Schematic top view of the dynamic glancing angle deposition (DGLAD) apparatus responsible for the oscillatory motion during sputtering (not in scale).

The Cr-Al-N ceramic coatings investigated in the present study were manufactured in a Plasma-HiPIMS 250 deposition plant (Plasma-LIITS, Campinas, Brazil) [8]. In this plant, both the sputtering process and negative bias tension are provided by Pinnacle DC power supplies, MDX model, from Advanced Energy. The chamber has two heating resistances mounted on opposite walls for substrate heating, each one counting with a thermocouple for temperature monitoring. The substrate holder also counts with a third thermocouple to improve temperature control. All coatings were deposited on an AISI 304 stainless steel disc. Before deposition, the discs were ground with 2000 grit sandpaper and polished using 6, 3, and 1 µm diamond suspension and colloidal silica, resulting in a substrate mean roughness of 3 nm. Next, the discs were cleaned in an ultrasonic bath with acetone for 5 min, and blow-dried. Then, inside the plant, sitting 65 mm apart from the targets, the disc surfaces were pretreated by an ion etching process using Cr^+^ ions generated by plugging a pure Cr target to a high power impulse magnetron sputtering (HiPIMS) power supply. The HiPIMS configurations for the ion etching were as follows: 600 W of average power, frequency of 104 Hz, t_on_ of 50 µs, and 900 V. To improve the energy of the impinging Cr^+^ ions, a bias tension of −800 V was applied to the substrate [23,24]. The whole process of ion etching took one hour with the purpose of promoting substrate cleaning and shallow implantation of Cr beneath the substrate to improve adhesion between the stainless steel and the coating. After the ion etching, the substrate received a Cr base layer to improve adhesion between the coatings and the substrate. Alloy targets with compositions 50% at Al/50% at Cr and 70% at Al/30% at Cr were used to produce two different sets of samples, and the Cr-Al-N coating manufactured for them were designated as 50/50 Cr_1−x_Al_x_N and 70/30 Cr_1−x_Al_x_N, respectively. A reference coating with composition equal to CrN was manufactured from the Cr target. All targets were manufactured by A.M.P.E.R.E Alloys, France, and had a purity of 99.95%. The deposition parameters used to obtain the ceramic coatings investigated in this study are listed in Table 1. The substrates were oscillated in a dynamic glancing angle deposition (DGLAD) setup, as described in previous work elsewhere [8], with a range and period of oscillation of −5°/+5°, respectively, and period “t” of 12 s, to verify the effect of this novel technique in fast oscillation motion (See Figure 1).

### 2.2. Coating Characterization

Top and cross-section images were acquired with the aid of a FEI Inspect-F50 (FEI, Eindhoven, The Netherlands) scanning electron microscope (SEM) equipped with a windowless silicon drift detector (SDD) for energy dispersive analysis (EDS) (Apollo X SDD, EDAX, Mahwah, NJ, USA). X-ray diffraction (XRD) analyses in the θ–2θ geometry were carried out using a Rotaflex Ru200B diffractometer (Rigaku, Tokyo, Japan) equipped with a rotative anode and Cu Kα radiation (1.5418 Å).

Coatings chemical compositions were evaluated by glow discharge optical emission spectroscopy (GDOES) depth profile analysis. The measurements were carried out using a GDA 750 high-resolution (HR) spectrometer (Spectruma Analytik GmbH, Hof, Germany) with a 2.5 mm diameter anode working in DC excitation mode (constant voltage-constant current mode). Triplicates were measured for each sample. The measurements were conducted under an inert Ar atmosphere (5.0 quality) and average discharge pressure of 5 × 10^−2^ hPa. The excitation parameters were 1000 V and 12 mA, with a sputtering rate for measuring depth of at least 75 µm. Profiles of mass concentration (%) vs. depth from atomic concentration (%) vs. depth were plotted using the WinGDOES.

Atomic force microscopy (AFM) measurements (area of 30 × 30 µm) in tapping mode were performed using a NanosurfFlex (Nanosurf, Liestal, Switzerland) to measure the coating surface finishing after deposition. The hardness of the coatings and elastic modulus were determined using instrumented nanoindentation tests at maximum normal forces of 50 mN with a PB1000 (Nanovea, Irvine, CA, USA) mechanical tester equipped with a Berkovich diamond tip. The indenter was calibrated using a fused silica standard. The Oliver and Pharr equations were used to calculate the hardness values [25]. At least 7 measurements were performed on top of each coating to determine an average value.

The sin^2^ ψ method with 7 ψ tilts was accomplished to measure residual stresses, for each sample (with sin^2^ ψ ranging from 0 to 0.9) [26]. Due to their high multiplicity, the (422), (511), and (333) diffraction lines of fcc-Cr_1−x_Al_x_ N were used for averaging the d hkl-sin^2^ ψ profiles in the stress analyses. A Panalytical MRD-XL (Panalytical, Almelo, The Netherlands) diffractometer equipped with Mo-Kα radiation (0.7093 Å) was used for all measurements.

The scanning transmission electron microscopy (STEM) images were produced using a JEOL JEM-2100 microscope (JEOL, Tokyo, Japan) equipped with a thermionic emission LaB6 electron gun, available at LNNano, Campinas, Brazil.

## 3. Results and Discussion

Investigations were conducted to understand the impact of fast substrate oscillation during dcMS depositions and also the effect of Al addition to coatings produced under N-rich atmospheres in different aspects of the resulting nitrides, to indicate whether or not they were suitable for hard coating applications. This section is subdivided concerning the impact of such modifications in different aspects of the coatings.

### 3.1. Chemical Composition and Microstructure

**Figure 2:**
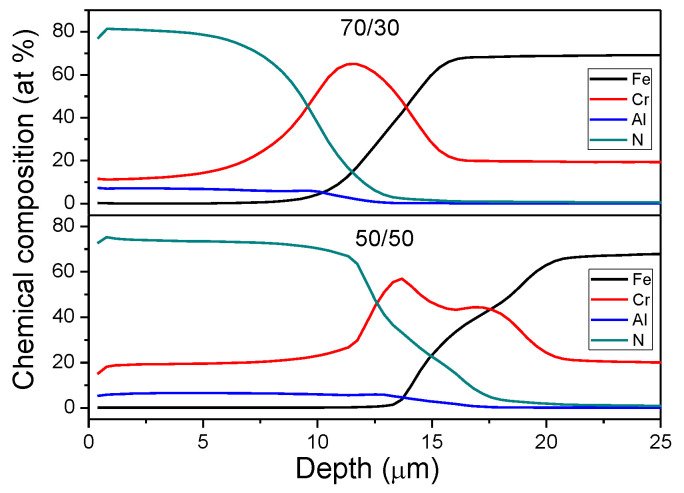
Glow discharge optical emission spectroscopy (GDOES) chemical depth profiling of the Cr_1−x_Al_x_N coatings.

EDS chemical analysis accomplished with a windowless SDD in the CrN coating revealed that its chemical composition was 49 ± 2 at.% N and 51 ± 2 at.% Cr, which is close to stoichiometry. The GDOES was used to evaluate the chemical composition (in-depth profiling) of the Cr_1−x_Al_x_N coatings manufactured with different Al/Cr precursor ratios, see Figure 2. In both Cr_1−x_Al_x_N coatings deposited, a high nitrogen content (~70–80%) was observed, which emphasizes its non-stoichiometric character. The manufactured coatings also had a higher concentration of Cr than Al even when produced using the 70/30 target.

Gas flow rates from 50 to 40 sccm of N_2_ and Ar, respectively, which have been proven to be adequate for deposition of stoichiometric CrN, produced N rich Cr_1−x_Al_x_N coatings. This could possibly be explained by the significantly higher sputtering yield of Cr as compared with Al [27]. Additionally, [28] attributed this lower sputtering yield of Al to the poisoning of the target surface by an AlN layer on the surface of the target. This becomes particularly relevant for large N_2_ partial pressures. Such a layer is promoted by the higher amount of Al in the 70/30 target and results in less material arriving from the target to the substrate. The preferential sputtering of Cr due to formation of AlN could possibly explain the small content of Al in coatings produced using the 70/30 nominal target.

A 50% increase in relative Cr content is observed from the 70/30 Cr_1−x_Al_x_N to the 50/50 Cr_1−x_Al_x_N coating. It is important to observe that at the same pace that Cr content increases, the atomic N concentration changes from around 80% in the 70/30 Cr_1−x_Al_x_N to 75% in the 50/50 Cr_1−x_Al_x_N. The difference in nitrogen concentration could also be an effect of the lower sputtering yield of the targets containing more Al (formation of the AlN layer). Therefore, with a lower flux of metallic particles arriving at the substrate from the target, the coating tends to be nitrogen richer. Nevertheless, in Figure 2, a broad Cr peak can be observed at approximately 12 µm for the 70/30 Cr_1−x_Al_x_N coating. This corresponds to the metallic Cr interlayer deposited previously on the nitride layer. In the case of the 50/50 Cr_1−x_Al_x_N coating, a double peak is noticed. This indicates that Cr could have been implanted in the substrate as a result of the pretreatment of the surface of the substrate with Cr^+^ ion.

In addition, the thickness of the coating can be estimated from the depth profile, since N and Al are meant to be present only in the nitride layer, with the base layer being richer in Cr. One can notice that there is a continuous non-abrupt interface between the two layers and that the Al richer 70/30 coating is roughly 2 microns thinner than the 50/50 coating, although both were deposited under the same deposition parameters.

**Figure 3:**
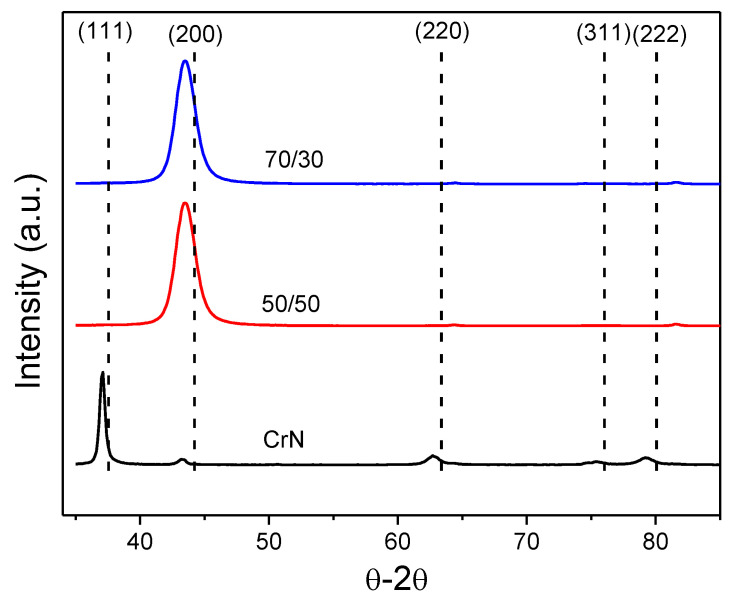
θ–2θ XRD measurements from coatings showing strong dependency on Al presence. Peaks position is related to the B1-CrN structure (ICDD 00-001-0065 reference).

Figure 3 shows the XRD diffractograms measured from CrN and Cr_1−x_Al_x_N coatings. Coatings containing Al presented sharp peaks related to the (200) plane of the CrN B1 cubic structure, in contrast to previous studies [14,22], in which no AlN or Al peaks were observed, even for the coating with a higher Al/Cr ratio. It was also evident that no other nitrides or oxides were formed, since the CrN cubic structure with Al solid solution was the only phase identified. In agreement with the reference position (dotted line) [29], (200) peaks were dislocated to smaller 2θ angles, which indicated shrinkage of the interplanar spacing (d). This was an effect of the presence of in-plane compressive residual stresses in the coatings [26]. In addition, the presence of Al contributed to the contraction of the lattice parameters, causing shifting in the CrN peak positions to lower than 2θ.

The XRD diffractogram measured from the CrN reference coating (see Figure 3) also indicated the formation of solely cubic B1-CrN with strong texture related to the (111) plane. The evident difference in texture between the Cr_1−x_Al_x_N and CrN can be explained by the lower sputtering yield of the alloy Cr-Al targets as compared with the Cr target. In the case of the former, while growing in an N-rich atmosphere, the film receives an arriving flow of predominantly N_2_ molecules and much smaller flux of Cr or Al neutrals or ions. Therefore, the formation of planes with higher N contents is expected in the surface, at the expense of metal-rich planes, which is the case of (200), in the CrN B1 structure. The lower deposition rate also favors the preferred orientation that minimizes surface energy [12], which is (200) in the case of CrN, making it even more favorable as the preferential orientation [30].

In addition, the CrN coating receives a higher flux of metal from the Cr pure target during growth as compared with Cr_1−x_Al_x_N, even for the same N_2_ flow rate, and develops texture of the type (111), which is a metal richer plane and also minimizes strain energy generated by the higher deposition rate [12,31].

### 3.2. Coating Morphology

**Figure 4:**
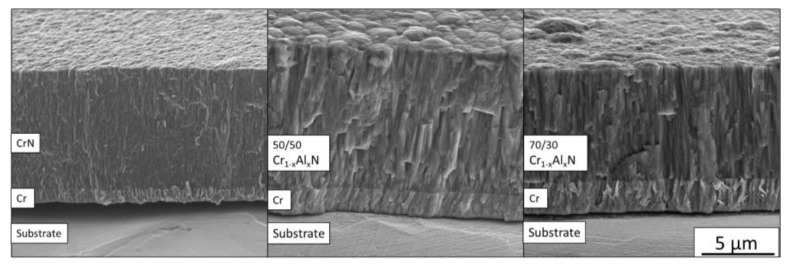
SEM images acquired from the cross-section view of the Cr-Al-N ceramic coatings. All images are in the same scale.

FEG-SEM cross-section images are shown in Figure 4. The CrN coating presents a structure characteristic of Zone II of a structure zone model (SZM) [31], with a homogeneously dense structure through its entire thickness. The 50/50 Cr_1−x_Al_x_N coating exhibits a less homogeneous structure, with thinner columns at the bottom of the film and “v” shaped grains at the top, which is characterized as a T Zone in the same SZM. The 70/30 Cr_1−x_Al_x_N coating, which is Al-richer, displays a similar morphology to that of the 50/50 condition, in a zone of dense but “v” shaped columnar grains.

**Figure 5:**
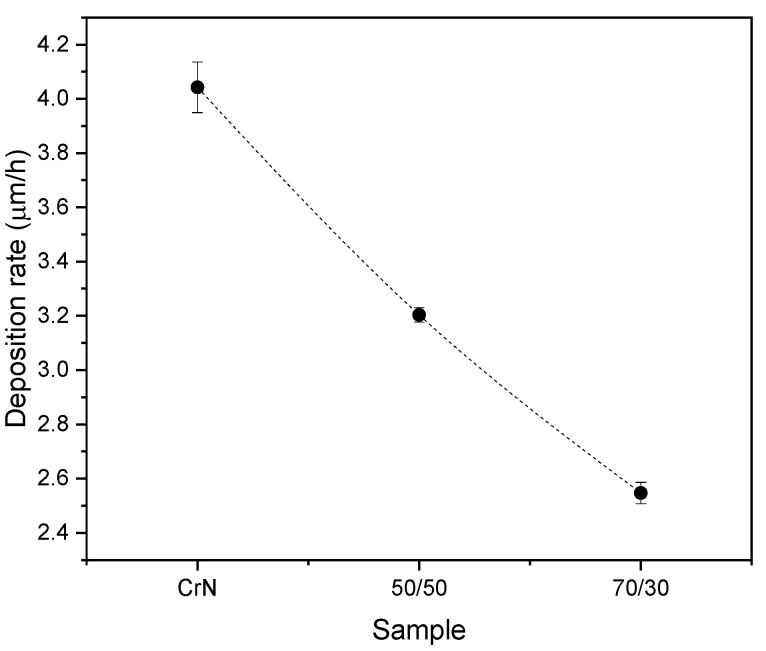
Deposition rate as a function of the Al/Cr ratio.

The average coating thickness follows the trend observed in the GDOES results, i.e., samples with higher contents of Al are the samples with the lower average deposition rate. The lower deposition rates visible in Figure 5 for coatings with a higher Al/Cr ratio also can be explained by the higher sputtering yield of Cr as compared with Al or Cr-Al alloys in reactive sputtering.

**Figure 6:**
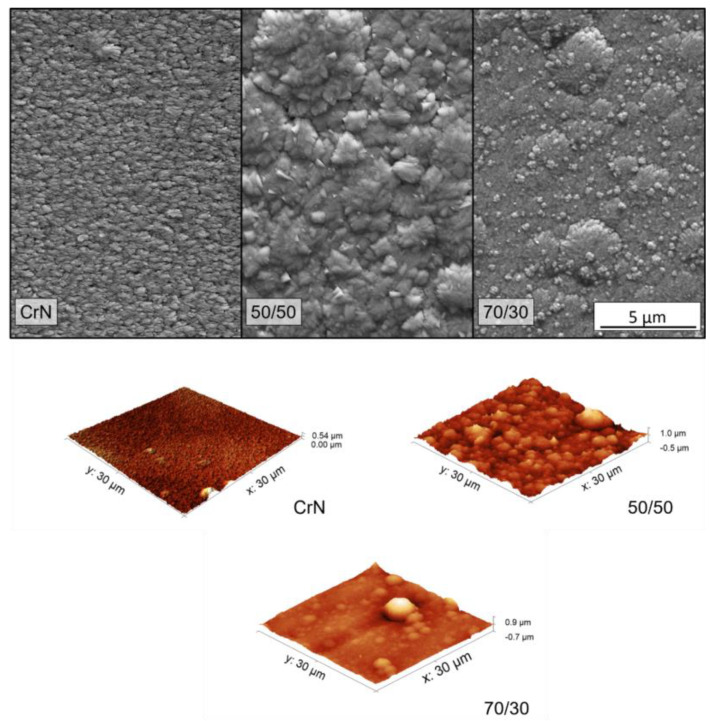
SEM images top view and atomic force microscopy (AFM) surface mapping of the Cr–Al–N ceramic coatings. All images are in the same scale.

The top SEM images and the AFM surface profile map are presented in Figure 6. All three coatings show dense surfaces with few pores or voids and densification increasing with Al content. The CrN surface presents a faceted pyramidal shape with some lateral facets, which is typical of nitride films grown with (111) preferential orientation [12,32]. As the Al was added, the grains’ top morphology change to round domes, which is also expected for a B1-type crystal textured in the (200) preferential orientation to decrease the overall surface tension [32]. These observations on the surface shape of grains confirm the texture change behavior observed in Figure 3.

In both Al-containing coatings, it was possible to see round morphological defects of a few microns distributed along the surface. These defects are inherent in the sputtering process and unavoidable to some level [33,34]. We observed that CrN presented finer grains and the addition of Al increased grain size. This was possibly due to the modification in growth morphology related to the change in texture. The 50/50 coating presented apparent larger grains with bigger domes, which are also visible in the cross-section images in Figure 4.

The effects of grain size and the presence of morphological defects have an impact on surface roughness. As illustrated in the surface maps in Figure 6 and presented in Table 2, CrN demonstrates lower roughness than the Al-containing coatings, with an increase of almost 40% in roughness as Al is added. This is due to the smaller dome size and an apparent absence of morphological defects, as observed in Figure 6. The 70/30 coating has a smoother surface than the 50/50 condition, due also to their difference in dome size.

**Figure 7:**
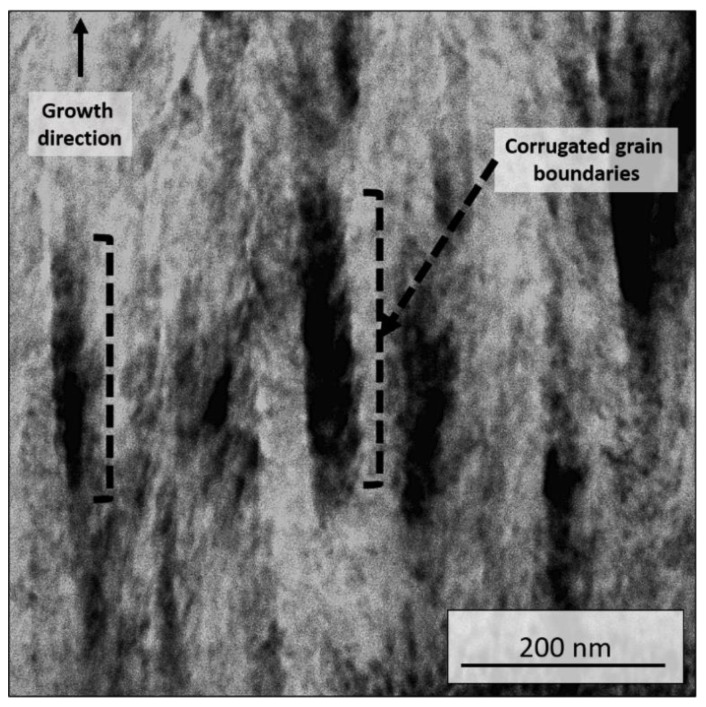
Bright field scanning transmission electron microscopy (STEM) image showing the corrugated nature of grain boundaries as a result of DGLAD growth in the 70/30 coating.

The effect of dynamic glancing angle deposition is demonstrated in Figure 7 for the 70/30 coating. As a result of the oscillatory motion of the substrate during sputtering within the range of +5°/−5° and period of 12 s, the columnar grains present a corrugated morphology at the boundaries (as exemplified in brackets in Figure 7). These corrugated zig-zag columnar grains are associated with in-grain misorientation due to differences in the angle of sputtering flux [6,8,35]. Figure 7 displays corrugated structures with periodicity ten-fold smaller, in the order of 30 nm, indicating that even fast oscillatory motions, with period as small as 12 s, are able to create such grain morphology. It was demonstrated by [8,31], and [36] that for coatings prepared by DGLAD, the properties were dependent on the period of oscillation, such as crystallite size, texture, and hardness. Therefore, these thin undulated features enhanced the performance of the coatings.

### 3.3. Mechanical Properties

**Figure 8:**
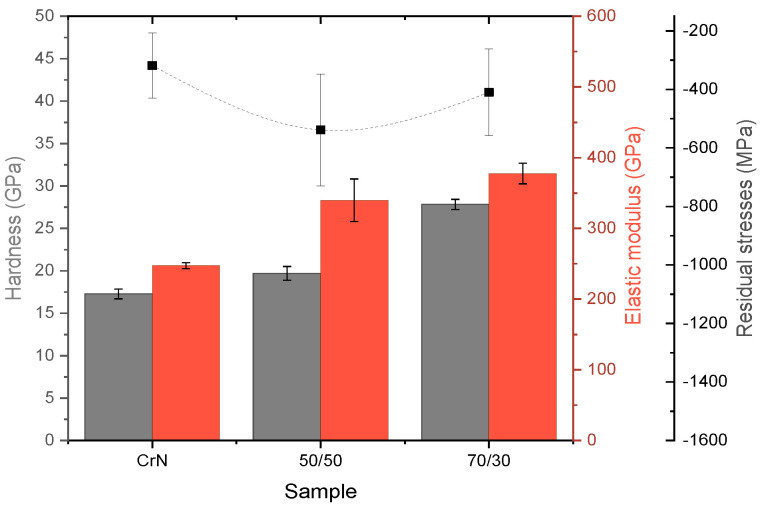
Hardness, elastic modulus, and residual stress as a function of the Al/Cr ratio.

The presence of residual stresses was confirmed by the XRD measurements performed using the sin^2^ ψ technique, see Figure 8. It shows the compressive stresses of hundreds of MPa as a result of the deposition process. On the one hand, the smallest compressive residual stresses were measured on the CrN coating. On the other hand, higher stress levels were measured on the 50/50 Cr_1−x_Al_x_N coating, which was slightly more significant than those observed for the 70/30 Cr_1−x_Al_x_N coating. Since all depositions were carried out using the same temperature conditions, power supply, bias, and chamber pressure, the same substrate heat was expected for all processes. Therefore, the difference in the chemical composition of the precursor target must be the only factor influencing the residual stresses on the films. The more significant compressive state of the Cr_1−x_Al_x_N coatings indicates that beyond the octahedral interstices normally occupied by nitrogen in a stoichiometric cubic CrN structure, the tetrahedral interstices in the lattice are also being occupied by the excess N, causing it to be strained [9].

Figure 8 presents the hardness and elastic modulus values measured by nanoindentation. In general, both hardness and elastic modulus increased with Al addition in the CrN coating. In this way, the highest Al/Cr ratio presented the highest hardness, with an improvement of around 37% as compared with the Al-free CrN reference. The behaviors are both in agreement with previous literature and can be explained by the solid solution hardening mechanism of Al in the CrN lattice [21,37,38]. For the sake of comparison, the bare AISI 304 substrate surface hardness was measured to be 3.4 ± 0.2 GPa, indicating that an expressive improvement in surface mechanical properties is achieved even for the Al free coating.

H^3^/Er^2^ is often related to resistance to plastic deformation [39]. Although the reduced elastic modulus (Er) does not take into account plasticity effects [40], it is a simple way to predict the coatings’ toughness and response to wear that requires no special sample preparation, especially for monolayer ceramic films. The H^3^/Er^2^ results are presented in Table 2. The film produced in the 50/50 Cr_1−x_Al_x_N condition presented the lowest value of H^3^/Er^2^, which could be an effect of the higher value residual stresses, as presented in Figure 8.

## 4. Conclusions

CrN and Cr_1−x_Al_x_N coatings with different Al contents were successfully manufactured using the DGLAD dcMS technique under an N-richer atmosphere. The DGLAD technique produced a multilayer-like architecture by growing corrugated grains with periodicity around 30 nm, indicating the capacity of this technique to tune the architecture of grains even at small scales. It was observed that the chemical depth profile was not influenced by the multilayer-like structure and, for the same deposition conditions, the CrN coating presented a chemical composition close to stoichiometric, whereas the Cr_1−x_Al_x_N films exhibited excess N, up to almost 80%, caused by a decrease in metal flux to the substrate as the Al-containing suffered poisoning, i.e., formation of the phase AlN on the surface of target. The deviation between the target precursor chemistry and the composition of the resulting coatings could also have been an effect of the target poisoning and a decrease in metal flux during deposition. The nitrogen enrichment changed the preferential growth orientation from (111) in CrN to (200) in Cr_1−x_Al_x_N. This change in texture caused the surface morphology of the coatings to go from a pyramid shape to a round dome form. The Al-richest coating presented the best mechanical performance, with higher hardness and resistance to plastic deformation as a result of solution hardening caused by Al. In this study, the presented results indicate that the combination of DGLAD and dcMS produce coatings with competitive mechanical properties as compared with techniques described in the literature, even in conditions of non-stoichiometry, and can serve as prospects for current industrial applications of hard coatings. A systematic variation of the substrate oscillation amplitude during dcMS appears to be a fundamental parameter to optimize grain misorientation within the coatings and further enhance the mechanical and tribological performance of single films with multilayer-like architecture.

## Figures and Tables

**Table 1:** Constant deposition parameters used to manufacture the CrN and Cr_1−x_Al_x_N ceramic coatings.

**Substrate Temperature**	400 °C
**Ar Flow**	40 sccm
**N_2_ Flow**	50 sccm
**DC Power**	900 W
**Negative Bias**	−120 V
**Working Pressure**	0.266 Pa

**Table 2:** Values of mean arithmetic roughness and H^3^/Er^2^.

Coatings	Mean Arithmetic Roughness Ra (nm)	H^3^/Er^2^ (GPa)
CrN	35.3	0.08117
50/50	129.2	0.07398
70/30	76.9	0.17868
